# Advancing reference emission levels in subnational and national REDD+ initiatives: a CLASlite approach

**DOI:** 10.1186/s13021-015-0015-8

**Published:** 2015-02-03

**Authors:** Florian Reimer, Gregory P Asner, Shijo Joseph

**Affiliations:** 1 Center for Development Research (ZEF), Group Börner, Rheinische Friedrich-Wilhelm University, Walter-Flex-Str. 3, Bonn, 53113, Germany; 2 Department of Global Ecology, Carnegie Institution for Science, 260 Panama Street, Stanford 94305, CA, USA; 3 Forest and Environment Program, Center for International Forestry Research, Jalan CIFOR, Bogor 16115, Indonesia

**Keywords:** Chocó, Colombia, Deforestation, Forest cover, Forest degradation, REDD+, Reference emissions

## Abstract

Conservation and monitoring of tropical forests requires accurate information on their extent and change dynamics. Cloud cover, sensor errors and technical barriers associated with satellite remote sensing data continue to prevent many national and sub-national REDD+ initiatives from developing their reference deforestation and forest degradation emission levels. Here we present a framework for large-scale historical forest cover change analysis using free multispectral satellite imagery in an extremely cloudy tropical forest region. The CLASlite approach provided highly automated mapping of tropical forest cover, deforestation and degradation from Landsat satellite imagery. Critically, the fractional cover of forest photosynthetic vegetation, non-photosynthetic vegetation, and bare substrates calculated by CLASlite provided scene-invariant quantities for forest cover, allowing for systematic mosaicking of incomplete satellite data coverage. A synthesized satellite-based data set of forest cover was thereby created, reducing image incompleteness caused by clouds, shadows or sensor errors. This approach can readily be implemented by single operators with highly constrained budgets. We test this framework on tropical forests of the Colombian Pacific Coast (Chocó) – one of the cloudiest regions on Earth, with successful comparison to the Colombian government’s deforestation map and a global deforestation map.

## Background

Reducing emissions from deforestation and forest degradation, and enhancing the carbon stocks (REDD+), remains a key strategy for mitigating climate change. Unlike many previous conservation efforts, REDD+ is constructed on the principles of additionality against a baseline or reference emission level (REL), with no displacement of emissions to neighboring areas (leakage). It is noted here that the United Nations Framework Convention on Climate Change (UNFCCC) and the World Bank’s Forest Carbon Partnership Facility (FCPF) use the term REL, while the Verified Carbon Standard (VCS) applies the term baseline. They are synonymous, as long as they are reported as greenhouse gas emissions in units of tons equivalent to CO_2_ (tCO_2_e). REDD+ intends to follow a hierarchical nested approach where project, subnational, and national initiatives contribute to the reduction in emission from deforestation and degradation. A consistent system that works across scales is therefore important for operationalizing REDD+, ensuring no displacement in the emission, and also to avoid potential double counting issues. The UNFCCC in its Warsaw Framework for REDD+ [1] specifies that such national forest monitoring systems “should provide data and information that are transparent, consistent over time, and are suitable for measuring, reporting and verifying anthropogenic forest-related emissions by sources and removals by sinks, forest carbon stocks, and forest carbon stock and forest area change”.

The role of remote sensing in measuring and monitoring forest area, and assessing its structural and functional attributes, has been well documented [2]-[4]. However, the REDD+ projects are often located in the humid tropics where a number of prevalent atmospheric and ground conditions, such as cloud cover, haze and uneven topography, often disrupt a satellite sensor’s ability to provide high quality observations of the land surface [5]. Moreover, spatial infrastructure, data access and technological expertise are key determinants of remote sensing capacity in countries within the tropics. This data limitation problem has been heavily reported and continues to be discussed [4]-[10]. Although operational monitoring of deforestation is reasonably possible with medium resolution remote sensing data such as Landsat, as evidenced from the Brazilian government’s program [11], establishing such a scheme at global scale is still underway. Progress has been made by the Global Forest Change program [12], and recent initiatives such as mapping of annual deforestation rate using Landsat data in the Congo basin [13], Sumatra [14], Colombia [15] and Peru [16] are also notable in this context. More importantly, such a system must make use of the satellite image pixel-based time series data compositing to minimize cloud, haze and other atmospheric artefacts that severely limit Landsat and other medium-resolution optical satellite data.

Here we report on the performance of a forest cover and deforestation mapping tool developed by Asner et al [17] for the operational monitoring of REDD+ landscapes in order to advance the readiness activities in carbon accounting frameworks. CLASlite is intended for a non-expert user to quickly assess the regional distribution of forest cover, deforestation and degradation. This makes it particularly appropriate for the establishment of sub-national to national reference levels in tropical regions with reduced satellite image quality and technical resources. CLASlite is intended to help the REDD+ community achieve rapid and reliable estimates of forest cover and deforestation. Here we test the efficacy of CLASlite in the context of new developments with sub-national and jurisdictional REDD+ initiatives. We also report on the performance of some new CLASlite modules such as the reduced masking option and deforestation artifact remover, and we elaborate on their effects on REDD+ reference levels and give recommendations for good practice. Furthermore we compare our mapping results with those of the Colombian national Institute of Hydrology, Metrology and Environmental Studies (IDEAM) [15] and of the new global maps recently made available by Hansen et al. [12].

### Study area

**Figure 1:**
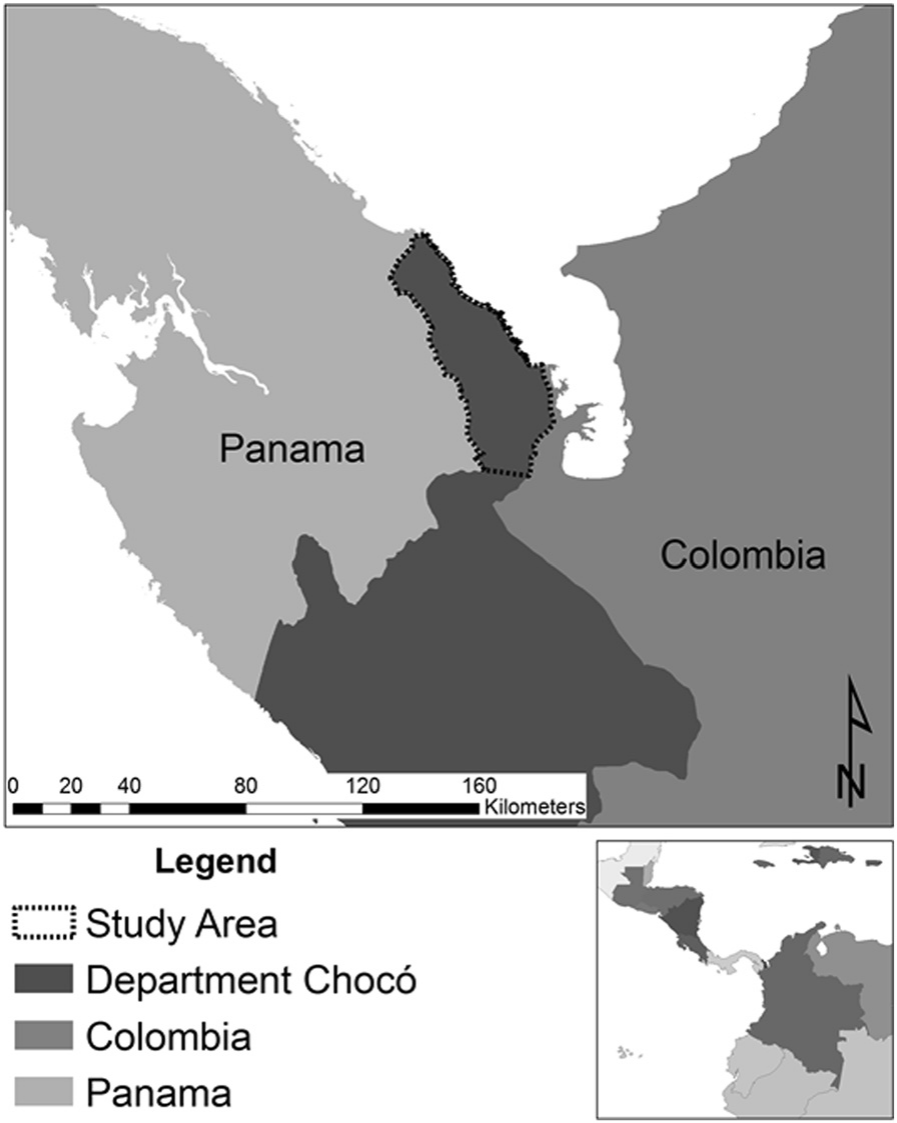
Location of the study region in Colombia.

The study region includes the Colombian municipality of Ancandi and the northern portion of Unguía, in the Department of Chocó. The southern border is formed by the common area coverage of Landsat image scene path-row 10 / 54 (Figure 1), with an area of about 1,900 km^2^. The approach presented here can be scaled up to regions of varying number of scenes and sub-national dimensions. 

Several municipalities form the northernmost portion of land of the Department of Chocó. They are bordered to the west by Panama’s Darién province and to the east by the Urabá Gulf of the Caribbean Sea, where the Rio Atrato forms a distinct delta known as “Bocas del Atrato” within the municipality of Turbo in neighboring department of Antioquia. Apart from logging, small-scale agriculture, fishery and cattle ranching, land-use in the study area includes illicit crops (*Erythroxylum* spp.) and activities around trafficking and contraband. Forests in the study region are exclusively humid Neotropical evergreen broadleaf in lowland, sub-montane, and pre-montane elevation ranges (1-1,400 m a.s.l.). Its peninsular geography at the Isthmus of Panama between the Caribbean Sea and the Pacific Ocean results in consistently heavy cloud cover. IDEAM estimates mean precipitation of 2,500-3,000 mm yr^−1^ in the region [18]. However, the Chocó harbors areas with > 9,000 mm of rainfall annually and is well known to be one of the rainiest regions on Earth. Studies suggest that the southern Department of Cauca (San Miguel de Micay) potentially has the highest recorded rainfall on Earth with an annual mean of 12,892 mm from 1971-2000 [18]. As a result, the Pacific coast of Colombia presents an extremely challenging case for optical remote sensing of forest cover and change. This challenge makes it an excellent laboratory to test new remote sensing approaches, and comparisons between monitoring systems can give us important information on the effects of monitoring design on mapped deforestation and therefore REDD+ reference emission levels [19].

### Results and discussion

#### Observed deforestation from mosaicked fractional covers

**Figure 2:**
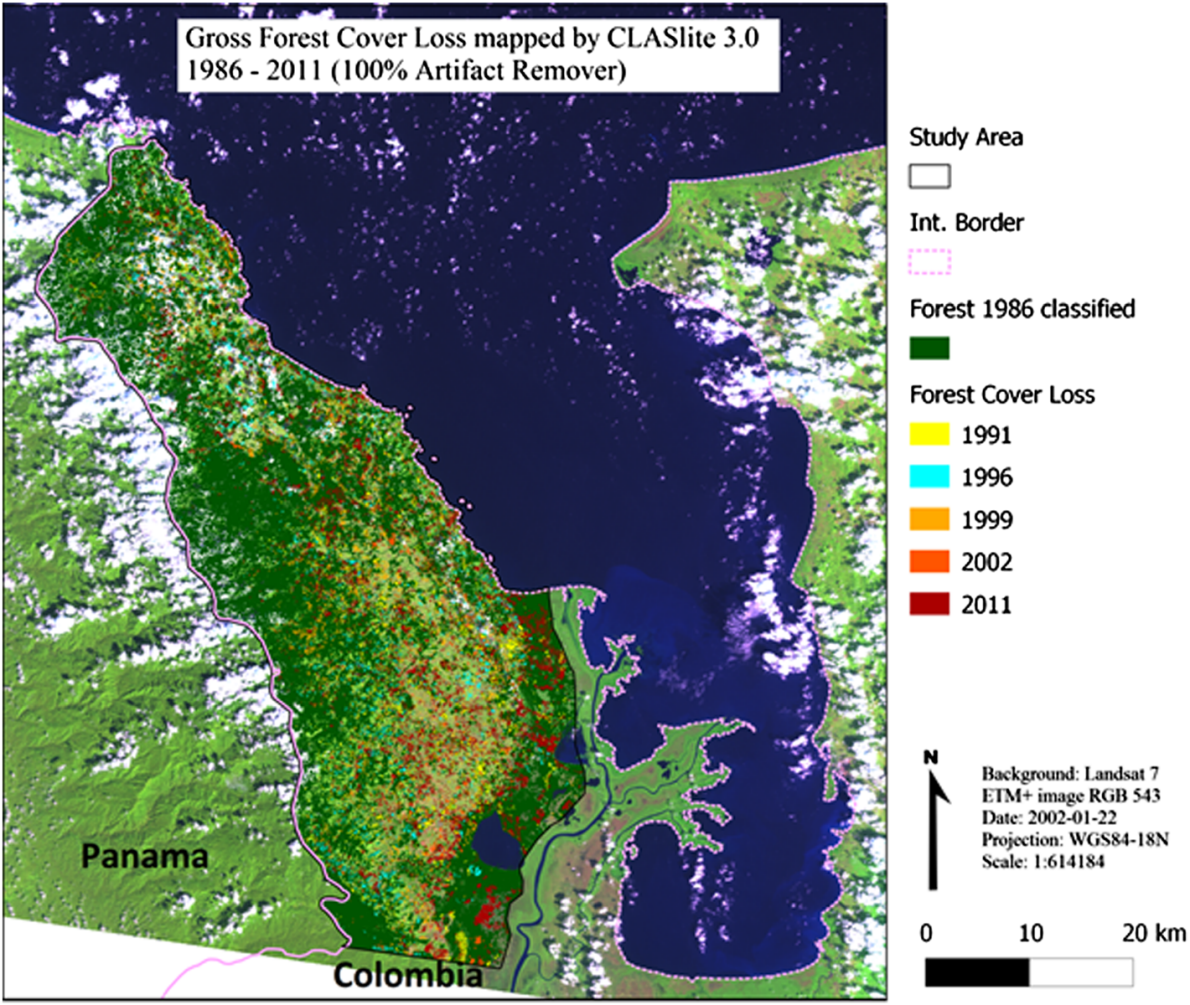
**Deforestation mapped by the most conservative option of 100% CLASlite v3.0 Artifact Remover.** Over a 25-year period, the region lost 30% of its original 1986 forest cover at a gross deforestation rate 1.210% yr^−1^.

The multi-temporal analysis covering 25 years (1986 – 2011) detected 30,681.3 ha of forest cover loss, which represents 26.31% of 1986 cover, or a deforestation rate of 1291 ha yr^−1^ (Figure 2). Such a long-term average rate is already applicable for a REDD+ REL, and can be extrapolated to the forest cover remaining at project start date [20]-[24] (Table 1). 

**Table 1:** Observed forest cover loss in study area by CLASlite v3.0 mosaic approach

**Time A**_**1**_	**Time A**_**2**_	**Forest cover A**_**1**_**[ha]**	**Forest cover A**_**2**_**[ha]**	**Def**_**period**_**[ha]**	**Deforestation rate yr**^**−1**^**[%]**
1986.049	1991.219	116,623.17	108,208.53	8,414.64	−1.45
1991.219	1996.520	113,263.83	109,904.49	3,359.34	−0.57
1996.520	1999.542	108,906.39	104,548.95	4,357.44	−1.35
1999.542	2002.498	106,949.25	104,992.11	1,957.14	−0.62
2002.498	2011.194	104,992.11	92,399.40	12,592.71	−1.47
**1986.049**	**2011.194**	**116,623.17**	**85,941.90**	**30,681.27**	**−1.21**

Rates of forest loss represent a convenient way of reporting deforestation in a globally comparable way [12]. However, there is little agreement among land-use change modelers as to whether projected deforestation rates should be predicted from average observed rates of forest cover loss or average observed rates of forest area lost. Using any rate of loss for prediction introduces problems, as the rate of loss not only depends equally on accurate mapping of the current area or cover, but also of accurate mapping of the original forest area or cover. In addition, rate of loss introduces a semantic problem: Researchers may quantify deforestation over large spatial and temporal extents, however, the process actually forms in local decision makers’ minds in terms of absolute areas, with no concept of the regional or national rate of forest loss. Simply speaking, farmers know how much land they need to clear for the expansion of a given activity, but they usually have little concept of how this area scales relative to the regional rate of loss. This may lead them to believe, if they have cleared 10,000 ha every year over the last 10 years, this quantity is the same as that converted regionally in the business as usual scenario. The land-holders do not know if those 10,000 ha cleared represent 1%, 0.1% or 0.01% of annual loss. REDD+ RELs predict emissions from forest carbon loss based on emission factors per activity type in Greenhouse Gas (GHG) emissions in tons equivalent to CO_2_ per ha (tCO_2_e ha^−1^). Therefore estimating emissions through predicted values of absolute forest area loss also in hectares per year is more straightforward and transparent than using a rate of loss in percent re-applied to remaining forest area. This is particularly true if the predicted future quantity of loss is not a stable average, but a function of the historic trend in quantities of loss [25].

#### Deforestation artifact remover test

**Figure 3:**
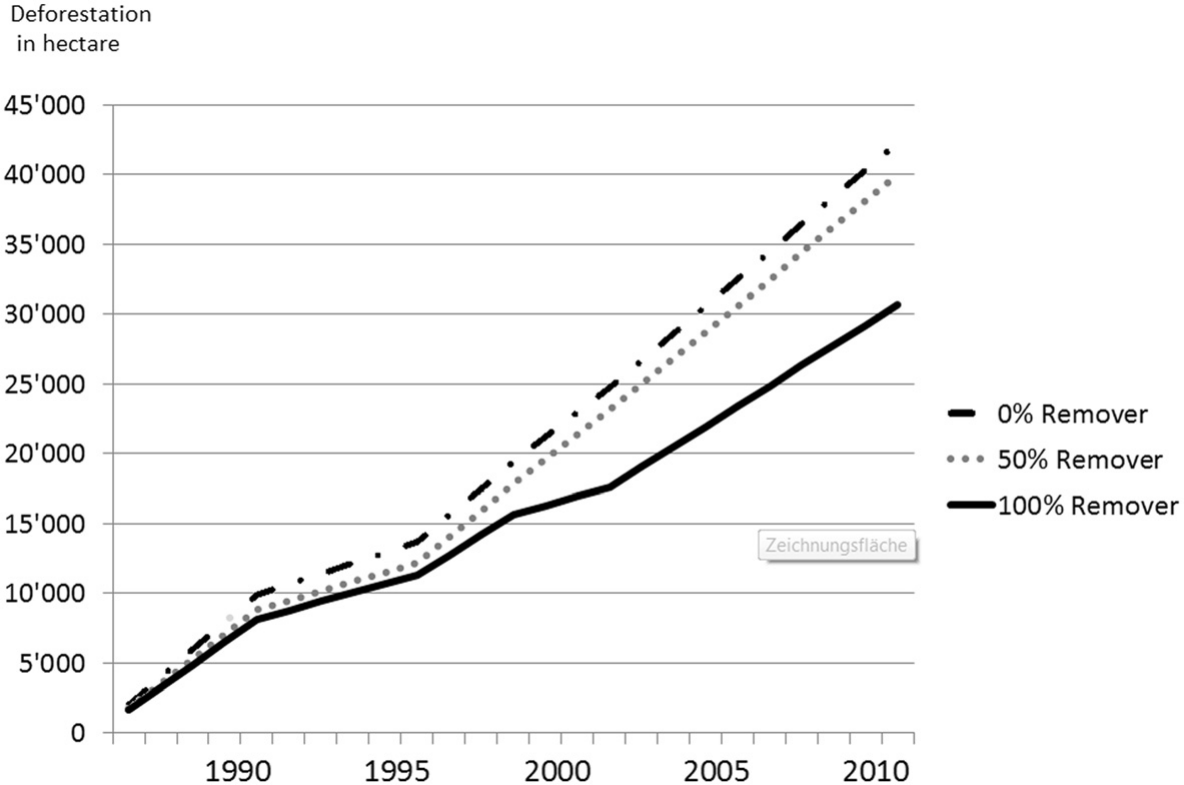
**Fractional cover images of the central scene in 2002 (left), auxiliary scene (middle) and mosaic (right).** Gaps could be filled especially in the south-east of the study area.

CLASlite 3.0 offers the user an option to apply a Deforestation Artifact Remover (DFAR) ranging in value from 0-100%. Unaltered forest change outputs may include unwanted artifacts (false positives) caused by the influence of clouds, unmasked cloud edges, cloud shadows, topography, and water boundaries. For Landsat imagery, the user can define desired settings for artifact removal in the deforestation image. In the standard operating mode, most artifacts are eliminated prior to analysis by the CLASlite pixel exclusion algorithm. With the user-selected DFAR value of 100%, CLASlite eliminates all pixels it recognizes as potential false positives. In contrast, at 0%, CLASlite does not eliminate any of these potential false positives. In order to assess the impact of this tool the time-series of mosaicked fraction cover images was run with the parameter set at DFAR values equal to 0%, 50%, and 100%. The accumulated deforested area over the monitoring period with a DFAR value of 0% was 137.6% of the area under 100% (Figure 3). 

As a general rule, all measurements, assumptions and models used in carbon projects like REDD+ should be “emission reduction conservative” [26]. This means, if a choice of methods and parameters is to be made between equally justifiable approaches, the preferred option should result in more conservative estimates of GHG emission reductions attributed to a climate change mitigation action in the end. Historical forest cover change monitoring is an essential element of the reference emission level of REDD+. Net GHG emission reductions creditable to a climate change mitigation intervention are calculated as a difference between REL and observed emissions from forest cover change (Measure, Report, Verify – MRV) in years of project operation [1],[20]-[24],[26]. This means that options should be selected to report conservative historical forest cover change to avoid risks of inflating the baseline emissions by measurement decisions.

Therefore, for a final report of historical deforestation, we choose the most conservative DFAR 100%. We also recommend this as the default for CLASlite 3.0 monitoring efforts with the aim to generate data for a REDD+ REL. Deviation by users from this conservative approach should be justified. This study does not analyze the forest degradation output of CLASlite 3.0. Should CLASlite 3.0 also be applied to generate data for REDD+ REL credit, it seems prudent to apply the same conservative option of 100% to the “Degradation Artifact Remover”.

#### Mosaicked vs. single scene time-steps

Our study also included a comparison of deforestation monitoring using a mosaic of multiple scenes versus single-scene per time-step input approach to CLASlite 3.0. We sought to determine if the new, mosaicked approach significantly increased the change area monitored in the observation periods and if the detection under both approaches is valid. To this end, the individual fractional cover maps of the central scenes were analyzed in a multi-temporal forest cover change analysis with the same default thresholds for the CLASlite 3.0 decision tree [27].

The single-scene approach, using only the central scenes with the same time steps as described earlier, thus following a conventional CLASlite processing, managed detected 45.59% (13,988.3 ha) of the deforestation mapped from 1986-2011 by the mosaicked fraction cover scenes (30,681.3 ha). In line with the principle of conservativeness [26], the most conservative option, 100% for DFAR was used. In all time steps, the mosaicked scene approach produced more forest cover change than the single scene approach – which is likely due to the addition of artifact-free pixels integrated from additional scenes to the observation area.

The mosaicked scene approach not only added 54.41% to the absolute forest cover change of the single scene approach, but also showed good spatial overlap with the single scene change results. Spatial overlap per time-step ranged from 37.05% (1991-1996) to 94.36% (1996-1999), averaging 62.43% over the entire observation period. This still leaves a substantial portion of single scene change results that are not picked up by the mosaicked approach.

We also analyzed how additional detected areas of forest change in the filled mosaic pixels might influence the change results. The additional forest change from the mosaicked approach, located in areas previously without data in the single-scene approach, varied from 7.52% (1986-1991) to 74.42% (2002-2011). On average, additional pixels in the mosaic approach resulted in a 39.11% in forest cover change over the single scene approach.

A detailed visual inspection of both forest cover outputs with the original Landsat imagery indicated a high probability of the mapped deforestation to be valid in both approaches. Therefore we conclude mosaicking fractional cover images, can aid in the assessment of forest cover in a greater proportion of the pixels allowing better detection and quantification of deforestation in environments of very low image quality due to persistent cloud cover or even sensor failures (Landsat 7 ETM+ SLC-off).

#### Comparison with previous work

For the study region, two independent deforestation datasets were available, one from IDEAM [15], the other from the University of Maryland, UMD [12]. A comparison of deforestation output from different remote sensing approaches can help to quantify the impact of monitoring approaches on estimated RELs [19]. REDD+ emission reductions are only useful if they are achieved relative a realistic REL, and quantification of historical deforestation is a central element to REL construction. In absence of historical, spatially extensive ground data, which are almost never available, it is not possible to verify or falsify the three datasets we compare here [26],[28],[29]. Instead, we compare the predicted range of REL values calculated from the deforestation results over the years 2000-2012 from the three different approaches, and we compare the predicted range of reference emission levels.

**Figure 4:**
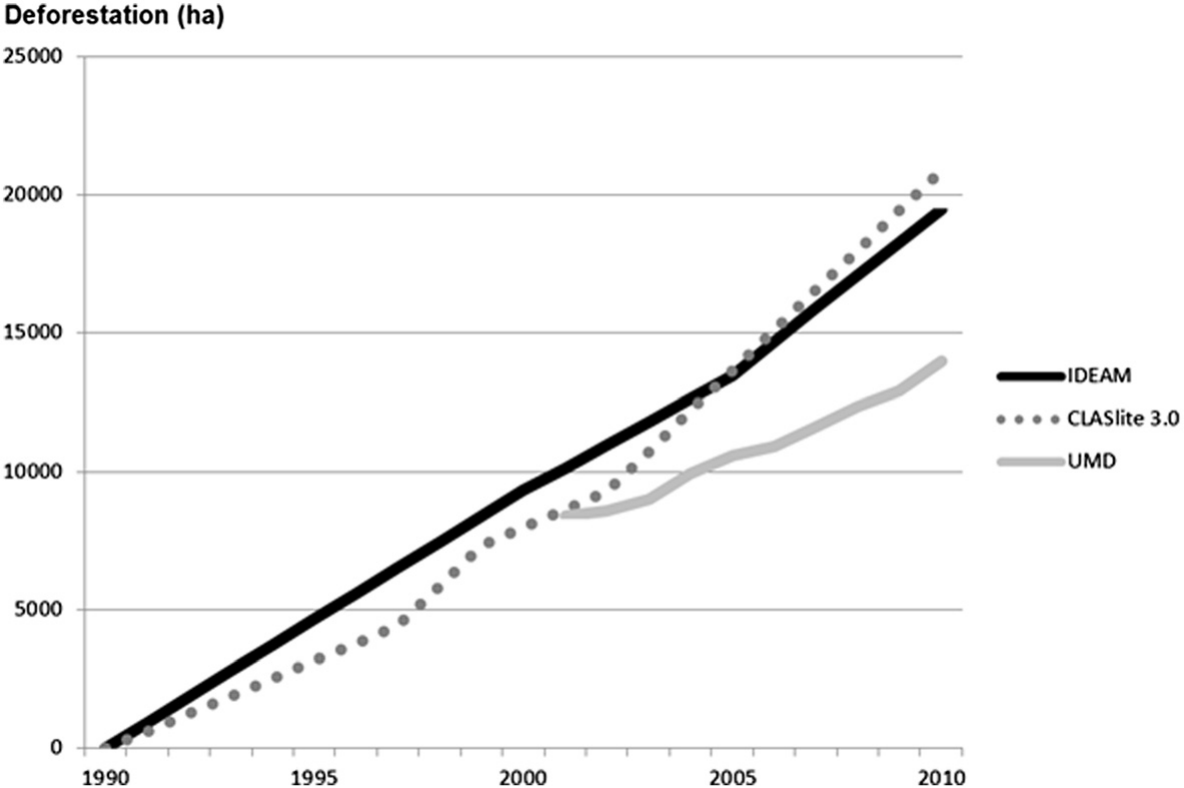
Accumulated deforestation in hectares, analyzing the same fractional cover mosaics with different parameters for the “Deforestation Artifact Remover” of CLASlite 3.0.

The three deforestation datasets covered different observation periods, possibly complicating the interpretation of the results. The study presented considered the longest period of 25 years from 1986-2011. The IDEAM dataset [15] covered 1990-2012, while UMD only covered [12] 2000-2012. To look at long-term temporal dynamics, we compare the three datasets in the 1990-2010 period in terms of accumulated deforestation per hectare (Figure 4). In the case of UMD [12], we incorporate the deforestation output of the CLASlite analysis of 2000 (lower compared to IDEAM) to allow for graphical comparison (Table 2). 

**Table 2:** Annual reference emission level calculated from three different deforestation datasets

	**Emission factor [tCO**_**2**_**e/ha]**	**Average loss 2000-2011 [ha]**	**Annual REL [tCO**_**2**_**e]**
IDEAM	500	1,019.23	509,615
CLASlite	500	1,288.95	644,473
UMD	500	603.24	301,619

Overall, IDEAM exhibited the lowest temporal variability and mid-levels of accumulated loss. Our results using CLASlite demonstrated moderate temporal variability and the highest accumulated loss, while the UMD method had the greatest temporal variability due to the annual temporal resolution and the lowest levels of accumulated loss. When the resulting imagery was visually inspected, the UMD method generally under-estimated deforestation losses compared to losses resulting from the CLASlite or IDEAM approaches. For IDEAM [15], the historical average rate of forest area loss was 1019.2 ha yr^−1^ between 2000 and 2010. Using CLASlite v3.0 with 100% artifact remover, the rate was 1288.9 ha yr^−1^, and for UMD the calculated loss was 603.2 ha yr^−1^. None of the three datasets indicated a clear increasing or decreasing trend in annual forest loss, so no regression was warranted [20].

We quantified the impact of the three deforestation datasets on the REL establishment. For simplicity we applied an average emission facto of 500 tCO_2_e ha^−1^ for each hectare of deforestation representing a single land-use change from a uniform forest to agriculture. Earlier we referred to the principle of conservativeness for GHG emission reduction quantification to justify our choice for the set of strict CLASlite v3.0 parameters that usually result in the lowest deforestation values. One might consider choosing the UMD approach for its extremely low historical average forest loss rate compared to the CLASlite and IDEAM output. However, such a decision would ignore two critically important factors:

a) The CLASlite mosaic-approach and IDEAM approach show a great similarity. Although the two datasets switch in terms of accumulated deforestation mapped in 2005, their overall accumulated outputs are, after 20 years, different by only 6.23%. Both approaches indicate an accumulated deforestation of about 20,000 ha in the period of 1990-2010 (20,890 ha CLASlite vs 19,506 ha IDEAM).

b) The UMD dataset detects less than 50% of the output mapped 2000-2010 by the CLASlite mosaic-approach, and just 56% of the IDEAM mapping for the same period. Remembering that we are dealing with a region with chronic cloud and shadow cover, it is possible that the global-scale automated UMD technique is not able to detect much of the deforestation in the region. Our visual inspection of the satellite data strongly supports the hypothesis that, in areas where IDEAM and CLASlite map deforestation, UMD misses true change visible in the raw satellite imagery.

The UMD approach uses a Landsat composite image from the greenest pixel calculation provided by Google Earth Engine [30]. We reviewed the Landsat greenest pixel product of [30] for the study region, and evaluated its use as a basis for the image mosaic approach. We have found that using the greenest NDVI or other metrics for image or pixel-scale mosaicking such as with the Google product severely reduces the amount of apparent forest cover lost over time, and in some regions it vastly over-estimates forest recovery. Moreover, the automated approach may include blurry and noisy data in the Google composites. Manual selection results in a much more consistent and reliable set of image inputs for use in deforestation and forest degradation detection and monitoring over time.

The UMD dataset is a very interesting experiment in automated global landcover mapping and change detection. It sparked a lively scientific discussion where it drew much acclaim and some criticism [31], including responses from the authors [32]. It provides an understanding of the general trajectory of forest cover change in any given country without differentiating between natural forest and plantation cover changes, while the later might include tree energy crops such as oil palm. This lack of ability to differentiate between natural old growth forests and plantation is true for all three approaches compared here [12],[15],[17]. The results can be viewed as indications of relative changes in forest dynamics (e.g. comparing the deforestation rate of an earlier period to a later period), but it the use to actually map absolute rates of old growth forest cover change for REDD+ purposes should be considered with caution and local interpretation.

All automatic land cover classification products can be validated for recent time steps by ground truth data in the form of confusion matrices [28], but ground truth data was not available to this study. For land cover & land use change products referring to periods several years in the past, map validation remains challenging. In a few instances, historic high resolution imagery such as aerial photography can be applied, but generally periods further in the past coincide with a lack of high resolution imagery and systematically sampled ground truth data.

#### Outlook for subnational RELs

REDD+ continues to be a concept in active development, and it has substantially evolved from a vague idea of “payments for forest carbon sequestration and storage” to real tests, for example in the Noell-Kempf Project [9] or the Kariba REDD+ Project [33]. REDD+ is taking a prominent role in international climate change mitigation negotiations [1],[22], and continues evolve through the development of a variety standards certifying REDD+ projects (CCBS, VCS, PlanVivo, ACR, CAR [34],[35]). The latest progress in this field was the publication of the VCS Jurisdictional and Nested REDD+ Requirements [21], the Warsaw UNFCCC decisions on REDD+ [1], and the FCPF Carbon Fund Methodological Framework [24]. On the other side, the first embryonic developments of compliance carbon trading schemes accepting and actively supporting international REDD+ offsets [34] are taking shape with the integration process of the system of payments for ecosystem services in the Brazilian state of Acre and the California compliance carbon offset and trading scheme. The link between the two could potentially be a verification of Acre’s GHG emission reductions by [21], and an acceptance of this approach into the California system.

From a REDD+ perspective, it should be noted that applying a REL built on forest carbon emissions has profound implications for whether a performance-based conservation program is adequately compensated. A REL built on an inaccurately low deforestation rate poses significant risks. For example, if due to underreporting, the REL only captures 50% of the average historic loss, a comparison with the REL built on underreporting would show no emission reduction against a true 50% decrease today. This would reduce or eliminate any real emission reduction based on REDD+, thus preventing financial resources from being allocated to a successful emissions reduction activity. The efficient allocation of financial resources to the most cost-effective climate change mitigation actions is a key rationale for REDD+, MRV and performance based payments [1],[3],[9],[22],[24],[26],[28],[34].

The role of NASA’s Landsat mission continues to stand out as primary data source for tropical historic land cover and land use change analysis, although the variety of sources is ever expanding and barriers to satellite data access are decreasing over time. Without entering an in-depth analysis on reasons for Landsat’s dominance, certain factors likely play a role: free access, easy catalog search, long-time continuity and a broad body of scientific research supporting the use of Landsat. However, the failures of Landsat 7 ETM+ SLC instrument 2003 and of Landsat 5 TM late 2011 have left data gaps in many areas until the new Landsat 8 became operational in June 2013. Approaches such as the framework presented in this study, to be implemented by single platform users or sophisticated cloud-computing approaches [16], can help to bridge these gaps, reduce uncertainties in historic observations and standardize the comparability of results by the application of the CLASlite modules for high quality image pre-processing and classification. This new approach can advance subnational REDD+ baselines and reference emission levels, considered crucial for verifiable emission reductions and forest carbon finance efforts.

## Conclusions

The proposed approach extends the traditional application of CLASlite for forest cover change monitoring and adds to CLASlite’s automation, speed, lowered technical barriers and mapping accuracy a new way to address incomplete imagery as a result of from clouds, shadows and sensor-failures. While this study only used automatic cloud and shadow masking of CLASlite, users can also easily decide on inclusion or exclusion of regions of interest, by manually drawn polygons (e.g. to mask smoke from fires distorting fractional cover values).

The proposed framework could support extended spatial and temporal observation coverage of a given region and monitoring period, where incomplete images and sensor failures limit spatial coverage and number of time steps. This may, in turn, contribute to increased validity, reduced workload and cost, and an inclusion of more time steps per monitoring period to capture forest cover change dynamics more aptly. This can also increase the fit of currently proposed regression equations to predict the quantity of future deforestation [20]. The mosaicked fractional cover scene approach can be a useful extension of observable area in time-series with the aim of detecting deforestation with CLASlite 3.0 when a user in a tropical region without cloud free season does not have access to more sophisticated approaches that select the greenest pixel per location [16]. The presented approach also supports experienced CLASlite users with limited resources who wish to expand their observable area in their time steps instantly with techniques already at their disposal.

Fractional cover values per pixel are products of a normalizing, yet iterative process that takes reflectance properties into account to find a best fit from spectral libraries. So, if not obscured by atmospheric phenomena missed by the cloud & shadow masking module (e.g. haze) or partial sensor failures (SLC-off pixel with values in some but not all 6 spectral bands), each pixel has the same validity whether coming from a July or March scene of the year of interest. This is mainly true for seasonal tropical forests, which are a common focus of CLASlite and our case study region.

A limitation to the proposed approach remains the subjective selection of a central scene and its auxiliary scenes to construct a meta-time step. This leaves the possibility that from the central scene not all sub-optimal pixels are masked and therefore are not replaced by mosaicking with the auxiliary scenes even if those would have clear-sky information at this location. Thus, information distorted by atmospheric phenomena (e.g. haze) potentially not accurately corrected in the modules of CLASlite would enter the final mosaic. Such sub-optimal pixels are avoided by more sophisticated approaches that select the greenest pixel per location using NDVI from all imagery taken of the year to generate an annual composite of maximum difference in photosynthetic vegetation (PV) between forests and non-forest land covers [27].

Calculating deforestation rates, we correct for the fact that central scenes are not the same acquisition date per year, by calculating an annual deforestation rate normalized for the number of days between time steps. We justify our recommendation to use absolute values of predicted area of deforestation and degradation for REDD+ REL development instead of rates.

As noted in earlier reviews of CLASlite deforestation outputs [19] and in the User Guide itself [27], the results of CLASlite – whether from a single-scene or multi-scene approach – should be carefully inspected and interpreted. Applying the principle of conservativeness for REDD+ projects – a manual subtraction of perceived false positive change results is always allowable, e.g. by vectorization of results and editing.

## Methods

### Landsat data availability

Freely available Landsat imagery from the United States Geological Survey was characterized by high cloud cover throughout the study region. Between 2002 and 2009, Landsat 5 Thematic Mapper (TM) imagery also had a time gap, leaving us with only Landsat 7 Enhanced Thematic Mapper (ETM+) data containing the Scan Line Corrector (SLC) error that occurred in 2003, rendering each image missing pixel data in stripes across the outer ~30% of each image. Mosaicking of multispectral imagery from different acquisition dates often brings changed radiance properties, however, various approaches have been developed and applied [36]-[38]. This leaves the user with the option to classify incomplete imagery separately, assess map accuracy separately, and later mosaic land-cover classification products. Such efforts with incomplete imagery require much more work, and are ultimately severely limited by a scarcity of valid ground truth data for classification training and for map validation. This incomplete imagery problem that plagues many large-scale multi-temporal monitoring efforts of forest cover change can be remedied by using CLASlite’s unique ability to calculate fractional cover values invariant from radiance value properties through standardized atmospheric correction and the iterative fitting of reflectance values to spectral libraries of typical fractional covers. Given the limited Landsat data availability for our study region, we selected a monitoring period from 1986 to 2011 – a 25 year period covered by six time steps (1986, 1991, 1996, 1999, 2002, and 2011), and therefore five sub-periods with deforestation (net forest change) analysis.

As the main advantage of the presented approach is a low barrier improvement to forest cover monitoring in highly clouded areas, it should be noted that other technologies, such as InSAR (Interferometric synthetic aperture radar) also hold great value for observation of forest cover in highly cloudy regions. Most prominently, the PALSAR (Phased Array type L-band Synthetic Aperture Radar) sensor on the Japanese ALOS (Advanced Land Observation Satellite) has been repeatedly applied for this purpose [39],[40], including the Pacific Coast of Colombia (Niels Wielaard, personal communication). Please also see section 2.9.3 of [26] for discussion of the technology in the context of REDD+. There are some qualifications to be made about InSAR based deforestation monitoring: Though not insurmountable, the processing of InSAR imagery requires an even more specialized expert-knowledge than the application of multi-spectral imagery in a semi-automated process such as CLASlite v3.0 already demands. Several factors such as terrain relief particularly pronounced in the Chocó department require careful correction in order to avoid distortions to land cover & land use change results. For the analysis of historic deforestation, the continuity of imagery time-series and free data availability is an important aspect, where NASA´s Landsat mission remains unmatched. Recognizing the great potential and contribution of InSAR applictions to tropical forest monitoring, we focus in this study on presenting a low barrier approach for users with limited resources.

### Image calibration in CLASlite

CLASlite contains an automated set of algorithms that converts Landsat and other satellite images from raw digital number (DN) recordings to final maps of forest cover and forest change (both deforestation and forest disturbance) [17]. CLASlite’s approach includes four major automated steps, and several minor yet important “bad-image” data masking steps [27]. First, the raw DN images are converted to top-of-atmosphere radiance images using sensor offset and gain values provided by the satellite data source (e.g. USGS for Landsat). Then the radiance images are converted to apparent surface reflectance using a combination of atmospheric correction with the 6S model [41] and, if needed, haze correction [18]. Within this step CLASlite 3.0 offers the a standard set of masking parameters, which use optical and thermal channels from Landsat to remove conservatively mask or remove portions of the image that contain atmospheric phenomena like haze, clouds, cloud edges and shadows, and topographic shade [17]. The user can select the “Reduced Masking” option, in order to decrease the area masked by altering the sensitivity of the optical and thermal channels to cloud and cloud shadow spectral signatures. In this study we found that the masking process widely avoided problems of deforestation over-detection, but also conservatively masked many pixels with apparently valid DN values for which a valid fractional cover calculation seemed reasonable.

For forest cover change, the original forest cover (1986 in our case) is important in order to map change from it. To maximize our chances of picking up valid forest cover change, we utilized the “Reduced Masking” option for the original forest cover of 1986, but the standard masking for the later time-steps of change detection – thus increasing our valid original cover, but mapping change still conservatively.

### Generating fraction covers by AutoMCU

Image calibration is followed by the most important step in CLASlite: the Automated Monte Carlo Unmixing (AutoMCU) algorithm [42],[43], which is applied on the image, providing the fractional cover of photosynthetic vegetation (PV), non-photosynthetic vegetation (NPV), and bare substrate (S) on a scale from 0-100% cover in every image pixel. Critical to this study, the AutoMCU employs spectral endmember libraries for PV, NPV and S that are derived from thousands of hyperspectral measurements made using field, airborne and spaceborne imaging spectrometers [17]. Because the PV, NPV and S spectral libraries already incorporate enormous variation in reflectance properties of land covers, including under widely varying atmospheric conditions, the probabilistic approach usually leads to a very stable result in each pixel, even if the data come from different sensors or times of the year (as long as the data are not heavily cloud or atmospherically contaminated). This provides leverage for compositing different spatial subsets of AutoMCU output to allow for mosaicking the clearest pixels throughout a region otherwise heavily contaminated by clouds over time.

The final step of CLASlite takes the outputs from the reflectance and AutoMCU (PV, NPV, S) steps, and applies a series of decision trees to estimate forest cover on single-date imagery and forest change on multi-temporal images [17]. The decision trees have been steadily expanded and improved to allow for multiple tropical forest types, from lowland to montane forests. These decision trees are mostly empirically derived from validation studies in the field, and by input from the CLASlite user community [27]. In total, 17 Landsat 5 TM & Landsat 7 ETM+ scenes were obtained and processed using the CLASlite approach.

### Mosaicking fractional covers

**Figure 5:**
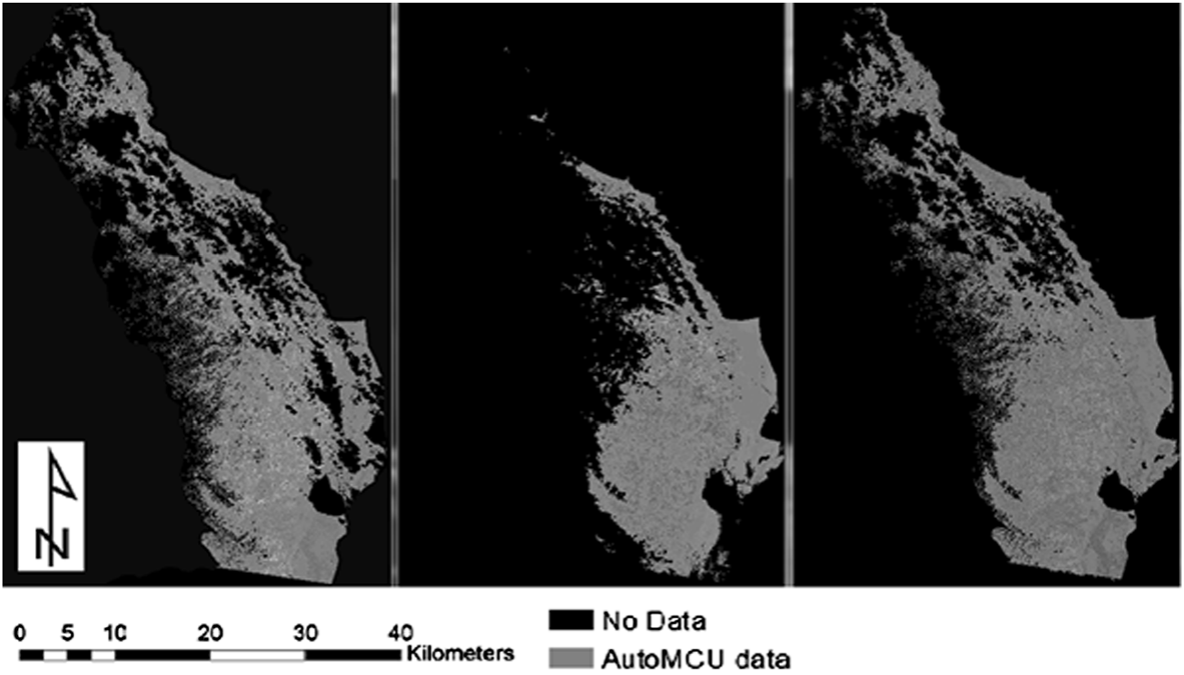
Comparison of accumulated deforestation output of the IDEAM, CLASlite v3.0 and UMD methogologies.

Mosaicking the individual fractional cover of CLASlite follows a minimal invasive approach to not distort DN values. The image identified as the best central scene of the time-step is used as top image, the other auxiliary scenes below. DN value -1 which represents masked areas in the fractional cover is used as “see through value” in order to allow fill-in from the auxiliary scenes. No color-matching is applied, so each valid fractional cover value per pixel entering the final mosaicked image is the same as coming out of CLASlite’s original processing. The results of increasing areas filled with multispectral reflection information is shown in Figure 5 below – base image left, auxiliary fill image middle and mosaicked image on the right. 

To define which images were candidates for mosaicking of their fractional cover images, rules were established to facilitate multi-temporal analysis. At each time step we identified a central scene of best quality. Additionally up to three other auxiliary scenes were selected within the temporal range of +/- 12 month around the central scene. Because forest types in the project area are evergreen, we assumed minimal seasonal effects on reflectance due to phenology. In addition, the spectral libraries within the AutoMCU sub-module of CLASlite allow for some degree of phenological variation in the spectra, with minimal effects on the fractional cover estimates.

### Estimation of deforestation rate

Under Decision 11/CP.7, the UNFCCC defined deforestation as: “the direct, human induced conversion of forested land to non-forested land.” This requires the application of a threshold between forested and non-forested land. We apply the forest definition reported by the Colombian Designated National Authority (DNA) to the UNFCCC CDM Executive Board:

 Minimum forest area: 1 hectare

 Tree crown cover value: 30%

 Tree height (or in situ potential to reach it): 5 meters

Deforestation rate is an important parameter to express deforested area comparable between all locations and scales. Puyravaud [44] suggested a standardized approach to calculate deforestation rates which has hence been widely applied, e.g. as baseline approach in [20], which can be applied to develop REDD+ RELs [21]-[23],[25]. To calculate an annual deforestation rate, it is necessary to adjust for the fact that satellite scenes per time step may not fall in the same month. A simple calculation using only years would look like:

Deforestation rate yr^− 1^ = {[1/(Year A_2_ – Year A_1_)] × log(A_2_/A_1_)} × 100, where:

A_1_ = Forest Area at beginning of time step

A_2_ = Forest Area at end of time step

Year A_1_ = Year of beginning of time step

Year A_2_ = Year of end of time step

This, however, could lead to distorted results as it would assume that a forest is always observed in the same month of each year, and thus the number of months between beginning and end of a time step implicitly being (Year A_2_ – Year A_1_) × 12. As is often the case, this is not the situation in our study, as the months of acquisition of our central scene varied from January to July.

Fortunately it is simple to include dates of image acquisition into Puyravaud’s [44] equation, adding the day count per year as a digit number. The exact day count is divided by 365.25 thus giving values ranging from 0.000 to 0.999. For example 17^th^ of July 1999 is translated to 1999.542. This way the difference between time A_2_ and time A_1_ accounts for the acquisition dates of the central image per time.

Deforestation rate yr^− 1^ = {[1/(time A_2_ – time A_1_)] × log(A_2_/A_1_)} × 100

where:

A_1_ = Forest Area at beginning of time step

A_2_ = Forest Area at end of time step

time A_1_ = Year and day count as digit number of beginning of time step

time A_2_ = Year and day count as digit number of end of time step

This topic becomes relevant, if for development of a REDD+ REL to project future deforestation from historic trends, rates of loss per year are used and not averages of absolute observed deforested area.

## Abbreviations

ACR: American Carbon AutoMCU: Automated Monte Carlo Unmixing algorithm

CAR: Climate Action Reserve CCBS: The Climate, Community & Biodiversity Alliance

CDM: Clean Development Mechanism

CLASlite: Carnegie Landsat Anlysis System lite

DN: Digital Number

ETM+: Enhanced Thematic Mapper

EU ETS: European Union Emissions Trading Scheme

FAO: Food and Agricultural Organisation of the United Nations

FRA: Global Forest Resources Assessments

FCPF: Forest Carbon Partnership Facility of the World Bank

GHG: Green House Gas

GIS: Geographical Information System

IDEAM: The National Institute of Hydrology, Metrology and Environmental Studies or Instituto de Hidrología, Meteorología y Estudios Ambientales de Colombia

INPE: INSTITUTO NACIONAL DE PESQUISAS ESPACIAIS – Brazilian National Institute of Space Research

MRV: Measuring, Reporting & Verification

NPV: Non-photosynthetic Vegetation

NDVI: Normalized Difference Vegetation Index

PlanVivo: Forest carbon community certification standard operated by the Plan Vivo foundation, a registered Scottish charity

PRODES: Project Deforestation (Projeto Desmatamento in port.)

PV: Photosynthetic Vegetation

REDD+: Reducing Emissions from Deforestation and Degradation and carbon stock enhancement

REL: Reference Emission Level

S: Bare Substrate

SLC: Scan Line Corrector

TM: Thematic Mapper

UMD: University of Maryland

UNFCCC: United Nations Framework Convention on Climate Change

USGS: United States Geological Survey

VCS: Verified Carbon Standard

OLI: Operational Land Imager

## Competing interests

The authors declare that they have no competing interests.

## Authors’ contributions

FR carried out the remote sensing analysis, designed the comparison approach and drafted the manuscript. GA has lead the development of the CLASlite software & approach, has given inputs on the remote sensing & comparison approach, provided literature indications and revised the manuscript. JS has given inputs to National & Sub-National REDD+ REL, provided literature indications and revised the manuscript. All authors read and approved the final manuscript.
